# Determinants of Digital Health Resource Utilization Among Individuals With Self‐Reported Bipolar Disorder in Germany—A Cross‐Sectional Study Based on the Andersen Model

**DOI:** 10.1111/bdi.70128

**Published:** 2026-06-09

**Authors:** Linda Kokwaro, Helena Krüger, Dennis Stratmann, Daniel Schulze, Daniel Fürstenau, Eva Friedel, Esther Quinlivan, Surjo R. Soekadar, Sonia Lech, Stefanie Schreiter

**Affiliations:** ^1^ Department of Psychiatry and Neurosciences Charité – Universitätsmedizin Berlin, Corporate Member of Freie Universität Berlin, Humboldt‐Universität Zu Berlin Berlin Germany; ^2^ Einstein Center for Neuroscience Charité – Universitätsmedizin Berlin, Corporate Member of Freie Universität Berlin, Humboldt‐Universität Berlin Berlin Germany; ^3^ Institute of Biometry and Clinical Epidemiology Charité – Universitätsmedizin Berlin Berlin Germany; ^4^ School of Business & Economics Freie Universität Berlin Berlin Germany; ^5^ Institute of Medical Informatics Charité – Universitätsmedizin Berlin, Corporate Member of Freie Universität Berlin, Humboldt‐Universität Berlin Berlin Germany

**Keywords:** Andersen healthcare utilization model, bipolar disorder, digital health resources

## Abstract

**Background:**

This study examines the utilization of digital health resources among individuals with bipolar disorder (BD) in Germany, applying the Andersen Healthcare Utilization Model (AHUA) to this context for the first time. While resources such as health‐related websites, social media and online forums, smartphone apps, digital devices such as wearables, and online interaction with health care professionals offer new opportunities for self‐management, less is known about which factors systematically influence their use among individuals with BD. To address this gap, the AHUA, which classifies healthcare utilization through predisposing, enabling, and need factors, was adapted to investigate digital health resource utilization.

**Methods:**

An anonymous online cross‐sectional survey of 213 individuals with a self‐reported diagnosis of bipolar disorder collected data on demographics, illness history, digital literacy, and digital health resource utilization. Hierarchical multiple linear regression analysis was conducted to examine associations between predisposing, enabling, and need‐related factors with digital health resources.

**Results:**

The final model explained 30.6% of the variance in digital health resource utilization. Among predisposing factors, female gender was associated with higher utilization. Among enabling factors, wearable ownership was the strongest and most significant factor associated with higher utilization. Among need factors, earlier age of diagnosis and lower health‐related quality of life were significantly associated with greater utilization. In contrast, smartphone ownership, digital health literacy, subjective health status, concentration difficulties, and the interaction between cognition and wearable ownership were not significantly associated with utilization.

**Discussion:**

These findings highlight that digital health resource utilization in individuals with BD is primarily driven by enabling factors and objective health‐related need rather than subjective health perceptions or cognitive functioning. The results underscore the importance of device access and clinical characteristics in influencing engagement with digital health tools. Targeting wearable‐based interventions and tailoring digital health solutions to individuals with greater clinical need may enhance adoption and improve self‐management in BD.

## 1 Introduction

Bipolar Disorder (BD) is a serious mental illness characterized by alternating phases of depression and (hypo‐)mania, affecting approximately 3% of the population worldwide. These recurring affective episodes can lead to significant morbidity, functional impairments, and an estimated 10‐year reduction in life expectancy [1]. Although pharmacological and psychotherapeutic treatment options remain the cornerstone of BD management, long‐term outcomes are often suboptimal, with many individuals experiencing residual symptoms, relapse or limited functional recovery [2]. Furthermore, access to care is often constrained by structural barriers, treatment gaps and long waiting times within mental health systems [3, 4, 5]. In recent years, digital health resources^1^ including emerging artificial intelligence (AI) based tools, have rapidly expanded and are increasingly integrated into mental healthcare systems [6, 7, 8]. These tools also hold potential for addressing gaps in conventional service delivery by providing flexible, cost‐effective, and scalable options for monitoring and therapeutic support [9]. Despite these promising avenues, individuals with BD face unique challenges when it comes to engaging with digital health resources [10]. For instance, manic or hypomanic episodes can lead to impulsive or inconsistent use of digital tools, whereas depressive phases may diminish motivation to initiate or sustain engagement [11, 12]. Concerns about data privacy and cybersecurity are also of great importance, especially in light of the sensitive nature of mental health data [13, 14]. During acute episodes, neurocognitive impairments, including shortened attention spans and slowed processing, can further complicate the use of interfaces that require sustained concentration [15, 16]. Digital health literacy poses another significant barrier. Many individuals lack the skills to navigate, assess, and apply health‐related content effectively, which may lead to misinformation or suboptimal outcomes [17, 18]. In addition, stigma and privacy concerns surrounding mental illness can deter individuals from participating in peer support forums or online therapy platforms, out of fear of being “outed” or labeled [19]. Finally, uneven regulation and quality assurance in mental health apps can create uncertainty about which tools are trustworthy and effective. For example, a review of the top 100 applications retrieved using the term “bipolar” in the iOS app store found that only 56% of the apps contained BD‐specific content, while most lacked peer‐reviewed evidence for efficacy [20]. Similar findings have been reported in analyses of the German Apple app store [21], highlighting ongoing concerns regarding the quality and clinical validity of available tools. In light of this and given the increasing uptake of digital mental health resources, it is essential to better understand patterns of digital health utilization. Healthcare utilization has long been used as a key indicator of the accessibility, effectiveness and responsiveness of healthcare systems [22]. Conceptual models, such as the Andersen Healthcare Utilization Model (AHUA) provide a structured framework for examining how individuals engage with healthcare services [23, 24]. Originally developed to explain the use of traditional healthcare services, the AHUA posits that healthcare utilization is driven by three determinants, namely: (1) predisposing characteristics (e.g., demographic factors such as age and gender), (2) enabling factors (e.g., income, healthcare coverage, digital health literacy) and (3) need factors (e.g., severity of illness) that encompass both evaluated (clinical) and perceived (subjective) health needs [23]. Within this framework, variables are classified conceptually based on their role in influencing healthcare use. Other studies on the AHUA expand predisposing factors to include social structure factors such as education, socioeconomic status, and health beliefs, while enabling factors are expanded to cover personal and contextual resources that facilitate access to care such as income and health insurance [22, 23, 25, 26]. Need factors on the other hand, capture both evaluated (objective health status) and perceived (subjective health) need [27, 28, 29]. Although some variables, such as education, may conceptually overlap across domains, they are typically assigned to pre‐disposing factors according to established conventions in prior applications of the model, which treat education as part of social structure [23, 30].

In traditional mental healthcare utilization studies, the AHUA has been widely applied in the context of depression [31], homelessness [32] and BD [27, 33], consistently demonstrating how individual characteristics and structural factors interact to influence healthcare service utilization. However, to our knowledge, the application of AHUA in digital mental health, particularly in the context of BD, remains underexplored. Given that digital health resources represent an extension of healthcare engagement as opposed to a standalone domain, applying the AHUA to a BD context provides an opportunity to move beyond descriptive studies and systematically identify determinants of digital health utilization in this population. Patterns of health service utilization further highlight the importance of examining determinants of healthcare utilization using a theoretical framework. A study conducted in high‐income countries found that less than one‐third of individuals with mental health disorders in Europe receive adequate treatment, highlighting considerable unmet needs [34]. Moreover, in Germany, analyses from the German Health Interview and Examination Survey for adults (ages 18–79) indicated that sociodemographic and diagnostic factors like female gender or comorbidities, respectively, influence access to mental health services [35]. In mood disorders in particular, delays of up to 7 years between disorder onset and service engagement [35] underscore the need to identify factors that not only influence the use of traditional health services but also the uptake of emerging digital health resources. Furthermore, evidence from traditional mental healthcare supports the relevance of the AHUA. A scoping review conducted in the Netherlands applying this framework found that illness‐related factors (defined under need factors) such as disease severity and diagnosis were the strongest predictors of specialized mental healthcare use [36]. On the other hand, enabling factors such as income and urbanicity contributed less, suggesting potential inequities in access [36]. These findings highlight the importance of systematically examining how different domains of the AHUA influence healthcare utilization. However, it remains unclear whether these patterns translate to digital health contexts, particularly among individuals with BD, where digital health resources present an increasingly vital complement to traditional care.

### 1.1 Present Study

**FIGURE 1:**
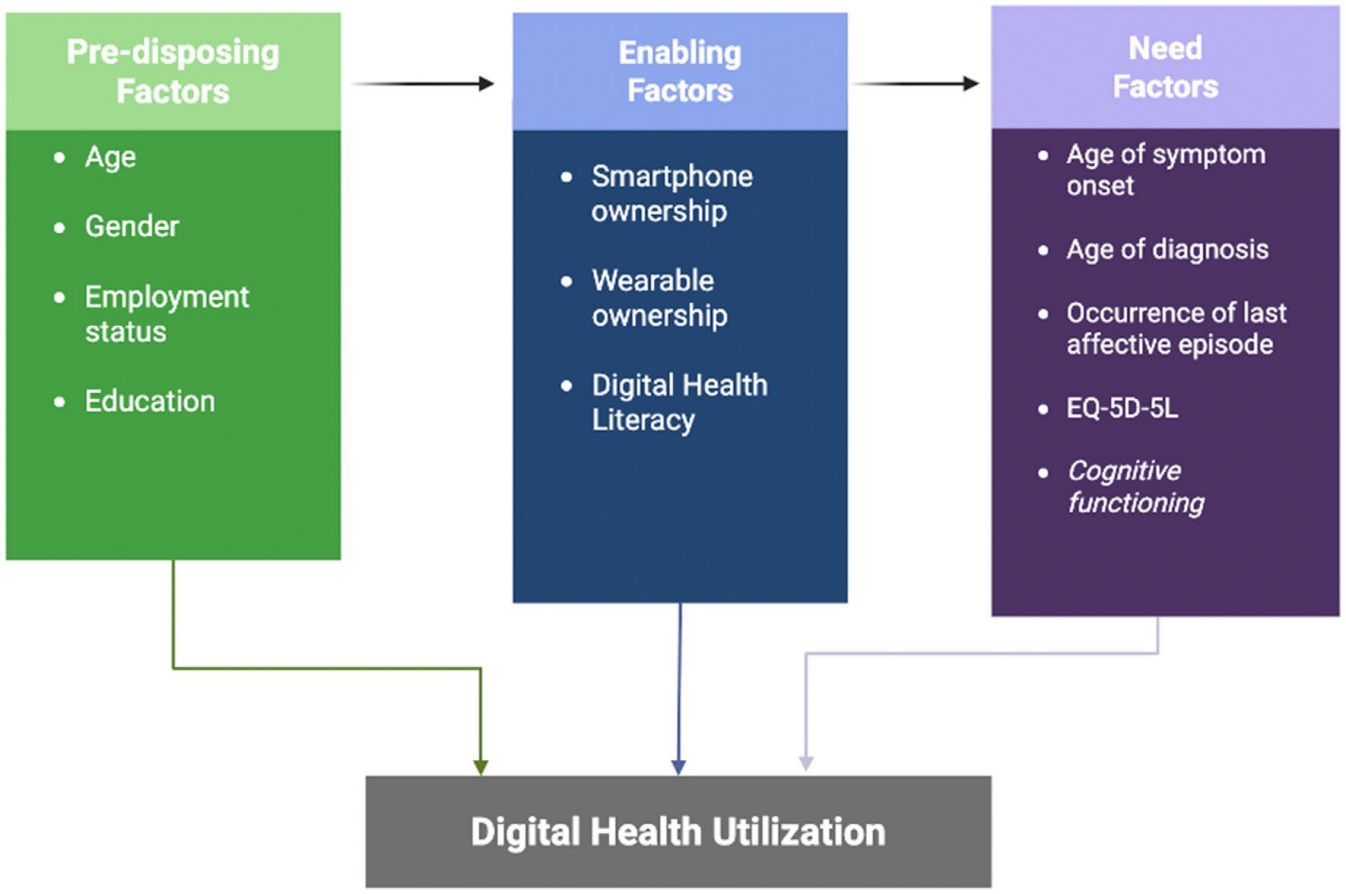
Conceptualization of the Andersen model of digital healthcare utilization among individuals with BD in Germany.

To address this gap, the present study applies the AHUA in a digital context to investigate patterns of utilization and identify key factors influencing the utilization of digital health resources among individuals with BD in Germany (Figure 1). Specifically, this study aims to (1) describe patterns of digital health resource utilization among individuals with BD, and (2) identify which AHUA factors, predisposing, enabling, and need, are most strongly associated with utilization in this population.

Based on prior theoretical and empirical work, we formulated three hypotheses. First (H1), certain predisposing characteristics (e.g., advanced age, lower education level, female gender, and employment status) will negatively influence digital health resource utilization. Second (H2), enabling factors, particularly smartphone/wearable ownership and strong digital health literacy, will positively influence digital health resource utilization. Third (H3), need factors, reflecting both evaluated and perceived need (i.e., subjective health status), including illness‐characteristics (e.g., age of diagnosis, age of symptom onset, recent affective episodes and cognitive functioning) and health‐related quality of life (EQ‐5D‐5L), are associated with digital health resource utilization, such that greater illness burden and lower health‐related quality of life will be associated with increased utilization.

## 2 Methods

### 2.1 Study Design and Setting

We conducted a nationwide, anonymous open online survey among individuals with BD between August 2023 and January 2024 in Germany. Ethical approval for this study was obtained from the Charité—Universitätsmedizin Berlin ethics committee (No. EA1/218/22). The survey was anonymous, allowing for increased participation, more open and honest responses and reduced social desirability bias, while enhancing privacy and confidentiality. To expand the reach and diversify the range of respondents, we engaged people who are part of (online) BD self‐help/support groups during the recruitment phase to create dissemination activities and materials. Subsequently, we leveraged already established connections through the Department of Psychiatry and Neurosciences at Charité with links to associations like the German Society of Bipolar Disorder and Bipolaris e.V., Pinel initiative (only organization that offers Ex‐In training for people with lived psychiatric experience).

### 2.2 Participants

For the quantitative survey, the study targeted a sample size of 250 subjects through convenience sampling. The inclusion criteria constituted a minimum age of 18 years and a diagnosis of BD (ICD‐10: F31). Participants were informed about the length of time of the survey, which data were stored and where and for how long, who the investigator was, and the purpose of the study. Informed consent was obtained prior to beginning the survey by clicking on a consent button which detailed the general terms and conditions of the study. Information on diagnosis was gathered through self‐report, and participants were additionally asked about who diagnosed them and the type of BD diagnosis.

### 2.3 Survey Development

The survey was co‐created with a group of 13 people with lived experience, peer specialists, and caregivers who took part in a 2‐day co‐creation workshop. The following bounds defined the inclusion criteria for the BD participants in the co‐creation workshops: minimum age of 18 years, an established diagnosis of BD (ICD‐10: F31), ability to give consent, present euthymic interval for at least 2 weeks or mild to moderate depressive symptoms (defined according to clinical cut‐off values in QIDS‐SR and ASRM), and sufficient knowledge of the German language. Conversely, the following exclusion criteria applied: acute suicidality, current severe affective episode or (hypo)manic episode (defined according to clinical cut‐off values in QIDS‐SR, ASRM), organic brain disorders, cognitive impairment, inability to understand the design and conduct of the study, as well as the associated risks and physical illnesses, which by their nature and severity could have interfered with the planned investigations or have an influence on the parameters to be investigated.

The participants, otherwise known as co‐researchers, were trained on the current state of research at the time, using quantitative surveys as a research method, and how to formulate appropriate questions and inclusive answer options using methods such as Likert scales to make sure that participants felt addressed and comfortable while taking part in the survey. Additionally, essential questions were identified and prioritized in addition to implementing adaptive questioning through conditional logic on some questions, which allowed for balance between data completeness and respondent comfort. The co‐researchers then tested the usability and technical functionality of the survey before it was fielded, and their feedback was incorporated into the final published version.

### 2.4 Variables and Instruments

The survey included sociodemographic and clinical variables and assessed digital heath care utilization and literacy; an overview of the assessed variables, measurements used and their classification according to the AHUA's factors is provided in Table S1.

The utilization of digital health resources was assessed using the 6 item HL‐DIGI‐DD instrument derived from the HLS_19_‐DIGI questionnaire, which is used to measure general digital health literacy und use within adult populations [37]. The HL‐DIGI‐DD instrument was used to assess subcategories such as (i) the use of health‐related websites, (ii) social media and online forums for health‐related content, (iii) digital health devices, for example, smartwatches, (iv) health apps for example, to monitor parameters such as sleep, (v) digital interaction with healthcare providers for example, online appointment scheduling, and (vi) the use of other general digital health resources. An example of a question was, “On how many days in a typical week do you use social media (including online forums) to find out or share information about health issues?” We additionally rephrased the general digital health‐related resource questions to focus specifically on bipolar‐related digital health resource use. An example of a question was, “On how many days in a typical week do you use social media (including online forums) to find out or share information about bipolar specific health issues?” The answer was measured using a 7‐point Likert scale ranging from less than once a week to having no experience at all. Respondents who stated that they use the digital health resources at least “less than once a week” were categorized as users. Respondents who stated “not relevant for me” were classified as non‐users. For the analyses, the respondents were then divided into “non‐users” and “users” [38], p2. The Cronbach's alpha for the scale was 0.64, indicating a moderate level of reliability. While this suggests that the six items may not fully capture digital healthcare utilization, the reliability is adequate for exploratory research or initial assessments, allowing for further refinement of the instrument in future studies. Additionally, Confirmatory Factor Analysis (CFA) was conducted to examine the underlying structure of the six items measuring Digital Health Resource Utilization. The analysis was first performed using the 2 different estimators: Maxiumum Likelihoof (ML) for robustness and Weighted Least Squares Mean and Variance adjusted (WLSMV) to account for ordinal responses. The CFA model demonstrated excellent fit indices, indicating that the six items collectively measure a single latent construct, Digital Health Resource Utilization (Tables S2 and S3).

#### 2.4.1 Digital Health Literacy

Digital Health literacy was assessed using a total of 10 items that are a part of the HL‐DIGI instrument, which included eight items on digital health literacy and two items to measure interaction with digital devices (HL‐DIGI‐INT reference). An example question was, “When you search for health information online, how easy or difficult is it for you/is it very easy, easy, difficult or very difficult for you to use the right words or search terms to find the information you need (online)?” The answer was measured using a 5‐point Likert scale ranging from very easy to having no experience at all.

#### 2.4.2 Bipolar Symptomatology

To evaluate current depressive and manic symptomatology the Quick Inventory of Depressive Symptomatology (QIDS‐SR) and Altman Self‐Rating Mania Scale (ASRM) [39, 40] were used. The QIDS‐SR assesses depressive severity with scores ranging from 0 to 27, interpreted as follows: 0–5 (no depression), 6–10 (mild depression), 11–15 (moderate depression), 16–20 (severe depression), and 21–27 (very severe depression). The ASRM measures manic symptoms on a scale of 0–20, with scores interpreted as 0–5 (no significant manic symptoms), 6–9 (mild to moderate manic symptoms), and ≥ 10 (high likelihood of mania). The Cronbach's alpha for the ASRM scale was 0.89, indicating a high level of reliability.

#### 2.4.3 Health‐Related Quality of Life

To evaluate health‐related quality of life, the EQ‐5D‐5L instrument, which is a patient‐reported outcomes instrument, was used [41]. It comprises a descriptive system that yields a utility‐based index score (EQ‐5D‐5L index) reflecting objective health status across five dimensions, as well as a visual analogue scale (EQ‐5D‐5L VAS) capturing an individual's self‐rated overall health on a scale from 0 to 100 [41]. In this study, the EQ‐5D‐5L VAS score was rescaled from 0–100 to a 0–10 metric for analysis. The index score was calculated using the German EQ‐5D‐5L value set, which applies country‐specific preference weights to the health state profiles [42].

### 2.5 Data Collection

The survey was disseminated across Germany through online and offline channels such as forums, social media, patient support groups, university and outpatient clinics with the additional help of co‐researchers. The survey was online between August 23, 2023 until January 07, 2024. Data was collected via Redcap.

### 2.6 Data Analyses

All statistical analyses were conducted using IBM SPSS Statistics (Version 29.0.2.0 (20)). Means and standard deviations were calculated, and comparisons between groups were conducted using standard tests such as *t*‐tests for continuous variables and Chi‐square tests for categorical variables. Distribution of continuous variables was assessed through visual inspection of histograms and the Shapiro–Wilk test. In case of non‐normal distribution, non‐parametric tests, such as the Mann–Whitney *U* test, were used for comparisons. Missing data was handled using pairwise deletion.

For the calculation of the HL‐DIGI‐DD score (digital health utilization), a mean score (ranging from 1 “Not relevant or less than once per week” to 5 “More than once per day”) was calculated as a relative measure for the frequency of use of health‐related digital resources [37]. Following this, assumptions for conducting a hierarchical multiple linear regression analysis were tested and met. Multicollinearity was assessed using variance inflation factors (VIF) and tolerance statistics, with all values within acceptable ranges (VIF < 5), indicating no evidence of multicollinearity. A hierarchical multiple linear regression analysis was then conducted to examine factors associated with the utilization of digital health resources, as operationalized by the HL‐DIGI‐DD score. Varaibles were entered in blocks reflecting the theoretical structure of the AHUA. In Step 1, predisposing factors (age, gender, employment status and education) were entered (Hypothesis 1). In Step 2, enabling factors (smartphone ownership, wearable ownership and the digital health literacy (DHL) score) were added (Hypotheses 2). In Step 3, concentration difficulties were included as a proxy for cognitive functioning. In Step 4, an interaction term between wearable ownership and concentration difficulties was entered to test for moderation effects. In Step 5, need factors (age of diagnosis, age of symptom onset, occurrence of the last affective episode and health‐related quality of life measured using the EQ‐5D‐5L index and the EQ‐5D‐5L VAS) were included (Hypotheses 3).

## 3 Results

### 3.1 Sample Characteristics

A total of *N* = 321 respondents clicked on the survey and *N* = 108 participants were excluded due to high missing values (ranging from 50%–100%), leaving a total of *N* = 213 participants who were included in the analysis. In the final analysis of 213 participants, 65.3% identified as female, 29.1% as male and 5.6% as diverse. The average age of the participants was 45.5 years (range: 18–78 years, SD = 12.5). The average age of symptom onset was 23.13 years (range: 5–62, SD = 11.3) and the average age when participants received their diagnosis was 35.38 years (range: 13–62, SD = 10.7), indicating a 12‐year gap in between. Regarding manic symptomatology as measured by the ASRM score (M = 2.6, SD = 3.6), 15.6% of the population had a score of 6 or higher, hinting that they had symptomatology towards a manic state. For depressive symptomatology as measured by the QIDS‐SR score (M = 10.9, SD = 5.3), 14.6% of the population had a score of 16 or higher, hinting towards a mild to moderate depressive symptomatology. Health‐related quality of life, measured using the EQ‐5D‐5L, showed a mean index score (M = 0.8, SD = 0.2), while the EQ VAS (10‐point) mean score was (M = 5.7, SD = 2.3). Most respondents were single (39.9%) and the majority lived in a large city (45.7%) with a population of more than 100,000 inhabitants. With regards to education, most respondents (61.5%) attained the highest German Highschool diploma (Abitur). The highest proportion of respondents were employed (39.9%) or in retirement (37.6%). For those who were employed, the majority were in part‐time employment (51.8%) and for those who were in retirement, the largest proportion (83.5%) was in early retirement with a reduced earning capacity pension. When asked about health insurance coverage, 90.1% of respondents reported that they are publicly insured, and the remaining 9.9% were privately insured. In terms of their current housing situation, 43.7% of respondents live together with their family or alone (39.4%) in an apartment or house. Detailed information on sample characteristics can be found in Table 1.

**TABLE 1:** Main sociodemographic and clinical characteristics of the sample.

Sociodemographic characteristics	Total sample, *n* (%) *N* = 213
Age: mean (SD, range)	45.5 (12.5, 18–78)
Gender	
Female	139 (65.6)
Diverse	11 (5.6)
Years of education: mean (SD, range)	11.5 (0.9, 9–12)
Marital status	
Single	85 (39.9)
Married	72 (33.8)
In a relationship	52 (24.4)
Distribution in Germany	
Rural area	41 (19.5)
Small city	24 (11.4)
Medium‐sized city	49 (23.3)
Large city	96 (45.7)
Health insurance	
Public	192 (90.1)
Private	21 (9.9)
Bipolar related assessments	
Type of bipolar diagnosis	
Type 1	103 (48.6)
Type 2	92 (43.4)
Other	12 (5.7)
Age of symptom onset: mean (SD, range)	23.1 (11.3, 5–62)
Age of diagnosis: mean (SD, range)	35.2 (10.7, 13–62)
QIDS‐SR score: mean (SD, range)	10.9 (5.3, 0–27)
ASRM score: mean (SD, range)	2.6 (3.6, 0–20)
EQ‐5D‐5L index: mean (SD, range)	0.8 (0.2, −0.4 to 1)
EQ‐5D‐5L VAS (10 point): mean (SD, range)	5.7 (2.3, 0.5–9.5)

*Note:* Values are presented as mean (SD, range) for continuous variables and *n* (%) for categorical variables. Missing data were handled using listwise deletion. The number of missing values for each variable did not exceed 5% of the total sample. Rural area (< 10,000 inhabitants), small city (10,000–20,000 inhabitants), medium‐sized city (20,000–100,000 inhabitants), large city (> 100,000 inhabitants). VAS, visual analogue scale, 10 point = the EQ‐5D‐5L VAS score was rescaled from 0–100 to a 0–10 metric for analysis.

### 3.2 Digital Health Utilization

**FIGURE 2:**
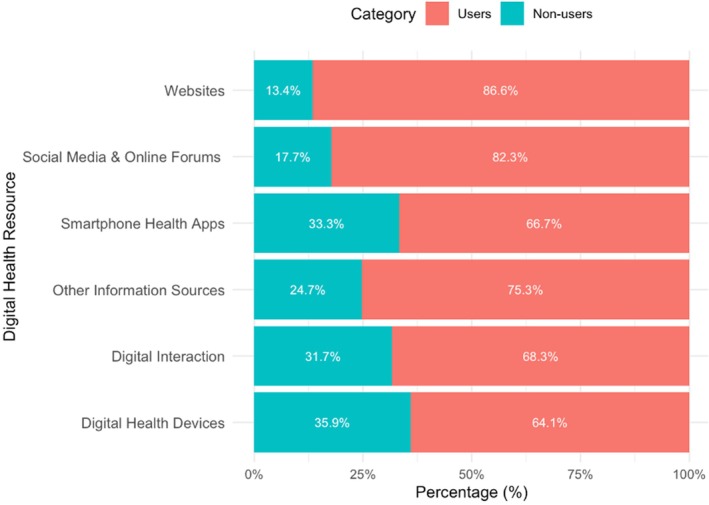
Distribution of users and non‐users of digital health resources.

The distribution of users and non‐users of digital health resources varied greatly, but overall, there were more users across each resource category as seen in Figure 2.

Among the respondents, 86.6% (*N* = 175) reported using health‐related websites as a digital health resource, while only 13.3% (*N* = 27) were classified as non‐users. Social media and online forums were accessed by 82.3% (*N* = 167) of participants, leaving 17.7% (*N* = 36) as non‐users. Similarly, 66.7% (*N* = 124) of respondents utilized smartphone health apps, whereas 33.3% (*N* = 62) did not engage with these tools.

When examining other information sources such as artificial intelligence chat bots and virtual reality, 75.3% (*N* = 137) of participants identified as users compared to 24.7% (*N* = 45) non‐users. Regarding digital interaction with healthcare providers, 68.3% (*N* = 123) reported engaging in such interactions, while 31.7% (*N* = 57) did not. Lastly, the use of digital health devices such as pedometers or fitness watches was reported by 64.1% (*N* = 123) of respondents, whereas 35.9% (*N* = 69) did not utilize these technologies.

**FIGURE 3:**
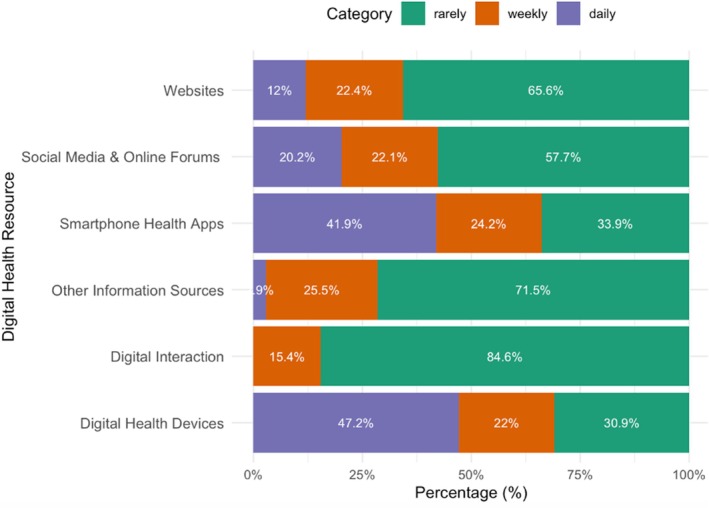
Frequency of use of digital health resources among users.

These findings, visualized in Figure 2, highlight a clear preference for digital health resources such as websites and social media while tools like digital health devices and smartphone health apps showed lower, yet substantial, levels of adoption. There were no significant differences between utilization of general and bipolar‐specific digital health resources (Figures S1 and S2). Following the categorization of users and non‐users, the frequency of use of digital health resources was then determined among users as seen in Figure 3.

The most frequently used digital health resource was digital health devices such as pedometers or fitness watches, with approximately 47.1% (*N* = 58) of respondents reporting daily use, 22% (*N* = 27) reporting weekly use, and 30.9% (*N* = 38) reporting rare use. Smartphone health apps followed as the second most used resource, with approximately 41.9% (*N* = 52) of respondents reporting daily use, 24.2% (*N* = 30) reporting weekly use, and 33.9% (*N* = 42) reporting rare use. Moderate frequency of use was reported for social media and online forums, with approximately 20.2% (*N* = 33) reporting daily use, 22.1% (*N* = 36) reporting weekly use, and 57.7% (*N* = 94) reporting rare use. Websites were moderately used among this sample, with 12% (*N* = 23) reporting daily use, 22.4% (*N* = 43) reporting weekly use, and 65.6% (*N* = 126) reporting rare use. Other information sources were used less frequently, with 2.9% (*N* = 4) reporting daily use, 25.5% (*N* = 35) reporting weekly use, and 71.5% (*N* = 98) reporting rare use. The least used digital health resource was digital interaction with a healthcare provider, with 15.4% (*N* = 19) reporting weekly use and the majority 84.6% (*N* = 104) reporting rare use. The average mean score of the HLS‐DIGI‐DD instrument for the utilization of digital devices was calculated as M = 1.29, SD = 0.79.

A hierarchical multiple linear regression analysis was conducted to examine factors associated with digital health resource utilization, operationalized by the HL‐DIGI‐DD score. Results of the final step (step 5) can be found in Table S4.

### 3.3 Predisposing Factors and Utilization of Digital Health Resources

In step 1, the associations between predisposing factors (age, gender, employment status, and education) and digital health resource utilization (HL‐DIGI‐DD score) were examined (Table 2). The model was statistically significant, adjusted *R* ^2^ = 0.064, *F*(4, 163) = 3.86, *p* = 0.005. Gender was significantly associated with the HL‐DIGI‐DD (*β* = 0.22, *p* = 0.01), whereby females reported higher utilization of digital health resources compared to males. Education was significantly negatively associated with the HL‐DIGI‐DD score (*β* = −0.16, *p* = 0.039), suggesting that individuals with more years of education reported lower utilization. Age and employment status were not significantly associated with digital health resource utilization in this step (Table 2).

**TABLE 2:** Hierarchical multiple linear regression model examining predisposing, enabling and need factors associated with digital health resource utilization.

Variable	Step 1		Step 2		Step 3		Step 4		Step 5	
	*B* [95% CI]	*β*	*B* [95% CI]	*β*	*B* [95% CI]	*β*	*B* [95% CI]	*β*	*B* [95% CI]	*β*
Model statistics										
*R* ^2^	0.09		0.22		0.23		0.23		0.31	
Δ*R* ^2^	0.09**		0.14***		0.01		0.00		0.08**	
Predisposing										
Age	−0.01 [−0.02, 0.01]	−0.07	0.00 [−0.01, 0.01]	−0.02	0.00 [−0.01, 0.01]	−0.01	0.00 [−0.01, 0.01]	−0.01	0.01 [−0.00, 0.02]	0.13
Gender	0.46** [0.13, 0.78]	0.22**	0.46** [0.15, 0.77]	0.22**	0.44** [0.13, 0.75]	0.21**	0.44** [0.13, 0.75]	0.21**	0.39* [0.08, 0.69]	0.18*
Employment	0.21 [−0.38, 0.80]	0.05	0.23 [−0.33, 0.78]	0.06	0.21 [−0.34, 0.76]	0.05	0.21 [−0.35, 0.76]	0.05	0.11 [−0.43, 0.66]	0.03
Education	−0.16* [−0.32, −0.01]	−0.16*	−0.16* [−0.31, −0.02]	−0.16*	−0.14 [−0.29, 0.01]	−0.13	−0.14 [−0.29, 0.01]	−0.13	−0.11 [−0.25, 0.04]	−0.10
Enabling										
Smartphone ownership			0.37 [−0.66, 1.40]	0.05	0.45 [−0.58, 1.48]	0.06	0.45 [−0.59, 1.49]	0.06	0.47 [−0.55, 1.49]	0.06
Wearable ownership			0.79*** [0.49, 1.09]	0.36***	0.81*** [0.50, 1.11]	0.37***	0.80*** [0.49, 1.11]	0.37***	0.89*** [0.59, 1.20]	0.41***
DHL score			0.00 [−0.00, 0.01]	0.02	0.00 [−0.00, 0.01]	0.03	0.00 [−0.00, 0.01]	0.03	0.00 [−0.00, 0.01]	0.05
Cognition/interaction										
Concentration					0.12 [−0.05, 0.29]	0.10	0.12 [−0.05, 0.29]	0.10	0.03 [−0.19, 0.25]	0.03
Wearable × concentration							0.00 [−0.38, 0.37]	−0.00	0.01 [−0.35, 0.38]	0.01
Need										
Last affective episode									−0.10 [−0.22, 0.03]	−0.12
Age of diagnosis									−0.02* [−0.04, −0.00]	−0.21*
Age of symptom onset									0.00 [−0.01, 0.01]	0.00
EQ‐5D‐5L VAS (10‐point)									0.07 [−0.02, 0.15]	0.15
EQ‐5D‐5L index									−1.26** [−2.13, −0.38]	−0.28**

*Note: N* = 168. Results of hierarchical multiple linear regression model. Cells report unstandardized coefficients (*B*) with 95% confidence intervals in brackets and standardized coefficients (*β*). *R* ^2^ = explained variance; Δ*R* ^2^ = change in explained variance. Values are rounded to two decimal places.

* *p* < 0.05.

** *p* < 0.01.

*** *p* < 0.001.

### 3.4 Enabling Factors and Utilization of Digital Health Resources

In Step 2, enabling factors (smartphone ownership, wearable ownership, and digital health literacy) were added to the model. This resulted in a significant increase in explained variance, Δ*R* ^2^ = 0.14, Δ*F*(3, 160) = 9.24, *p* < 0.001, with the model explaining 22.10% of the variance (*R* ^2^ = 0.22). Wearable ownership was significantly associated with digital health resource utilization (*β* = 0.36, *p* < 0.001), with individuals who owned a wearable device reporting higher utilization of digital health resources. In contrast, smartphone ownership (*β* = 0.05, *p* = 0.48) and digital health literacy (*β* = 0.02, *p* = 0.78) were not significantly associated with utilization after controlling for other variables (Table 2).

### 3.5 Cognitive and Interaction Factors

In Step 3, concentration difficulties were included as a proxy for cognitive functioning but did not significantly improve the model, Δ*R* ^2^ = 0.01, Δ*F*(1, 159) = 1.91, *p* = 0.169.

In Step 4, the interaction between wearable ownership and concentration difficulties was added to test for moderation effects. This interaction was not significant, Δ*R* ^2^ = 0.00, *ΔF*(1, 158) = 0.00, *p* = 0.977, indicating that the relationship between wearable ownership and utilization was not moderated by cognitive functioning (Table 2).

### 3.6 Need Factors and Utilization of Digital Health Resources

In Step 5, need‐related factors including age of diagnosis, age of symptom onset, last affective episode, EQ‐5D‐5L VAS, and EQ‐5D‐5L index were added, resulting in a significant increase in explained variance, Δ*R* ^2^ = 0.08, Δ*F*(5, 153) = 3.31, *p* = 0.007. The final model explained 30.60% of the variance in digital health resource utilization (*R* ^2^ = 0.31). In the final model, wearable ownership remained significantly associated with digital health resource utilization (*β* = 0.41, *p* < 0.001). Gender also remained significant (*β* = 0.18, *p* = 0.014), revealing higher utilization among females. Among need factors, age of diagnosis was significantly negatively associated with utilization (*β* = −0.21, *p* = 0.035), whereby individuals diagnosed at a younger age reported higher use of digital health resources. Additionally, the EQ‐5D‐5L index was significantly negatively associated with utilization (*β* = −0.28, *p* = 0.005) whereby lower objective health‐related quality of life was associated with greater utilization of digital health resources. In contrast, EQ‐5D‐5L VAS, age of symptom onset, and the occurrence of the last affective episode were not significantly associated with digital health resource utilization (Table 2).

### 3.7 Additional Findings

When participants were asked about whether they had used a program offered by their health insurance provider digitally, 80.6% (*N* = 170) answered no, while 19.4% (*N* = 41) answered yes. For those that reported using a digital health insurance program (*N* = 38), about half used a movement‐based program or one for relaxation and stress management. When asked whether they had installed an electronic patient record, 87.2% (*N* = 184) answered no while 12.8% (*N* = 27) answered yes. Participants were also asked about whether having experience with digital health resources made it easier for them to take health‐related decisions and the majority answered that this was partly true (42.2%, *N* = 89). For 14.7% (*N* = 31), this was not at all true; for 18.5% (*N* = 39), this was rather not true; for 19.9% (*N* = 42), this was rather true; and for 4.7% (*N* = 10), this was very true.

## 4 Discussion

### 4.1 Summary of Key Findings

The present study provides insight into the utilization of digital health resources among individuals with BD in Germany, highlighting the influence of predisposing, enabling and need factors which influence patterns of healthcare utilization. The main findings reveal that female gender (hypothesis 1), wearable ownership (hypothesis 2), an early age of diagnosis and lower health‐related quality of life (hypothesis 3) were associated with higher utilization of digital health resources. Notably, education showed a trend opposite to expectations that is, negative association with digital health utilization, suggesting that higher educational attainment may not necessarily translate into greater engagement with digital health resources in this population.

### 4.2 Utilization of Digital Health Resources

Predominantly, users outnumbered non‐users, with over 50% active users across all categories of digital health resources, indicating high utilization. Health‐related websites had the most users (86.6%), followed by social media and online forums (82.3%), while resources like digital health devices (64.1%) and smartphone health apps (66.7%) had substantial but relatively fewer users in comparison. Interestingly, among users, digital devices such as fitness trackers as well as smartphone health apps were used most frequently and had the highest proportion of daily users (47.2%). These findings differ from those of a study that investigated the use of digital health‐related information services among the general population in Germany, using the same HL‐DIGI‐DD instrument, where use varied among all resources but was overall lower than in the present study [38]. In the general population study, health‐related websites were the most frequently used resource weekly at 64.4% (in BD population: 86.6%), followed by social media (38.0%; in BD population: 82.3%). Across all categories, utilization in the BD population was higher than in the general population as seen further for categories such as digital health devices (31.5%; in BD population: 64.1%) and smartphone health apps (21%; in BD population: 66.7%). The most outstanding difference can be seen in the least used resource in the general population study, which was digital interaction at 15.9% compared to 68% in the BD population. This relatively high utilization in the BD population is further supported by the high rate of electronic health record use (12.8%), which is also significantly higher than in the general population of individuals with statutory health insurance in Germany, which stands at 0.9% [43, 44]. These findings also align with a study that investigated how personal and disease characteristics influence the adoption of electronic health records in Germany, where people with chronic illnesses were more likely than those with acute illnesses to use electronic health records because the perceived benefits were more important to them than for people with acute illnesses [45].

### 4.3 Application of the Andersen Healthcare Utilization Model

The AHUA was applied in this study to evaluate the influence of predisposing, enabling, and need factors on digital health resource utilization.

### 4.4 Predisposing Factors and Utilization of Digital Health Resources

Among predisposing factors in this study, gender was consistently found to be significantly associated with a higher digital health utilization, with females reporting higher utilization than males. This finding aligns with a report on the use of digital health applications among individuals with statutory health insurance coverage in Germany, which reported that females accounted for the majority of users at 71% [46]. Since the lifetime prevalence of BD is approximately equal across males and females [47, 48], the utilization patterns observed in this study point towards an underlying difference in help‐seeking behavior. Women tend to engage more in health‐seeking behaviors, including mental health apps, tracking symptoms and engaging with online communities [49, 50]. This trend corresponds with a prior study that reports that women exhibit higher digital health literacy and are more likely to seek online health information compared to men [51]. Understanding these gender‐specific preferences and behaviors is crucial for developing tailored digital health resources that effectively address unique gender‐specific needs. Contrary to expectations, education was not positively associated with digital health utilization and showed a trend in the opposite direction (H1). While higher educational attainment is typically associated with greater digital health literacy and increased engagement with digital health resources, evidence remains mixed, with several studies reporting no significant association, particularly in clinical populations [52, 53]. This finding suggests that education alone may not be a primary driver of engagement in this population. One possible explanation is that individuals with lower educational attainment may rely more heavily on accessible and low‐threshold digital tools to obtain health information and support, particularly in the context of barriers to traditional care. Conversely, individuals with higher education may have greater access to alternative sources of care or may be more selective in their use of digital health resources. Regarding age, there was no significant association with utilization in the final model, despite trends suggesting lower engagement among older individuals. While prior research indicates that older adults may face barriers related to digital literacy and access [54, 55] they may also demonstrate greater adherence once engaged [56].

### 4.5 Enabling Factors and Utilization of Digital Health Resources

Enabling factors played a central role in explaining digital health utilization. In particular, wearable ownership emerged as the strongest and most consistent factor associated with utilization across all steps of the model, highlighting the importance of device‐based access in facilitating engagement [57]. This finding suggests that wearables may act both as access points and as motivators for sustained interaction with digital health tools, for example through passive monitoring, feedback loops, and integration into daily routines [58, 59]. In contrast, smartphone ownership and digital health literacy were not significantly associated with utilization after accounting for other factors. This shows that having access to specific types of digital devices may be more critical in determining engagement than general digital competence. One possible explanation is that smartphone access has become widespread, reducing its explanatory value, whereas wearable devices may reflect a more active investment in digital self‐management. Important to note however, is that although recruitment was done primarily through online platforms, factors such as access to stable internet, and availability of tailored digital interventions were not directly measured but likely influenced utilization patterns. The present findings, however, suggest that not all forms of access are equally relevant, as enabling factors such as smartphone ownership and digital health literacy were not significantly associated with utilization, whereas wearable ownership emerged as a key factor. Evidence from traditional healthcare further supports the importance of structural determinants. In a study in the Netherlands which employed the AHUA to assess factors influencing specialized mental healthcare use, a greater contribution of social predisposing factors such as education and enabling factors such as income and urbanicity suggested inequity of the healthcare system [36]. It is therefore critical to address any covert systemic barriers to ensure equitable adoption of digital health technologies. For example, programs aimed at subsidizing device ownership, providing internet access, or developing culturally and linguistically tailored digital resources could be adopted to enhance inclusivity [60].

### 4.6 Need Factors and Utilization of Digital Health Resources

Among need factors, earlier age of diagnosis and lower health‐related quality of life (EQ‐5D‐5L index) were significantly associated with higher utilization of digital health resources. Notably, objective health status (EQ‐5D‐5L index) was associated with utilization, whereas subjective health perception (EQUATION 5D‐5L VAS) was not. This suggests that digital health utilization may be more closely associated with clinically relevant health burden compared to perceived well‐being. This distinction further reveals that individuals may engage with digital tools in response to measurable health needs instead of subjective evaluations alone. This finding aligns with recent applications of the AHUA, which posit that evaluated need is a primary driver of healthcare utilization [61, 62]. Furthermore, these findings suggest that individuals with greater objective health burden or longer experience managing their condition are more likely to engage with digital tools. In contrast, the age at which symptoms first appeared (onset) and the timing of the last affective episode did not significantly predict use of digital health tools. From a clinical standpoint, this is interesting because it suggests that early diagnosis, possibly coinciding with greater comfort in using technology, leads individuals to engage more with digital resources. An earlier diagnosis may allow individuals more time to accumulate knowledge, experience, and skills in managing their condition, which, in turn, facilitates engagement with digital health resources. If someone was diagnosed at an older age, they may not be as inclined (or have had as much time) to develop those digital self‐management habits. Clinicians might therefore want to pay special attention to older‐diagnosed patients who could benefit from digital tools but may need more guidance or education on how to use them. For patients themselves, it highlights how getting connected to digital supports early in the course of the illness might help normalize their use for long‐term management. Early identification would allow patients to access these digital resources sooner, facilitating symptom management, improving treatment adherence, and potentially reducing hospitalizations. In contrast, the age of symptom onset was not significantly associated with digital health resource utilization. A 12‐year gap was reported between symptom onset and diagnosis, which could indicate that although people may experience BD symptoms earlier in life, they only engage with digital health resources once they have an established diagnosis and consequently contact with the healthcare system. This corroborates findings from other studies that despite the substantial burden of illness, BD often remains undetected for approximately 7 years from symptom onset to diagnosis, resulting in lost opportunities for timely intervention [63]. Lowering barriers for early diagnostics or improving early detection through digital tools could have a positive impact on this gap. Interestingly, in the present study, digital interactions with health care providers had the lowest category of users, which could be related to the missing offerings.

Additionally, the occurrence of the last affective episode and concentration difficulties were not significantly associated with utilization of digital health resources. This suggests that episodic symptom fluctuations or cognitive factors may not substantially influence the perceived need for or use of digital resources, in comparison to broader and more stable indicators of illness burden. Due to the chronic nature of BD, the persistent need for management strategies irrespective of episodic severity could explain this finding. This could reflect a broader need for resources tailored to long‐term, rather than episodic, management applications for BD. Interestingly, a study in the Netherlands that applied the AHUA to assess factors influencing specialized mental healthcare use found that need factors, particularly disease severity, had a greater impact on utilization than demographic predisposing factors [36]. In this study, age and gender had a greater impact on utilization compared to illness‐related factors. Still, cognitive challenges or symptom severity, as suggested by prior research [64], might act as barriers to digital health engagement in certain individuals, further emphasizing the need for resources that accommodate varying levels of cognitive and emotional functioning. This will ensure accessibility and usability of digital health resources for people with lived BD experience [64].

### 4.7 Implications for Practice

These findings have significant implications for healthcare providers and policymakers. Digital health resource utilization among individuals with BD appears to be driven less by predisposing characteristics and more by enabling factors, particularly device‐based access and objective indicators of clinical need. In particular, the strong association between wearable ownership and digital health utilization highlights the potential of integrating wearable‐based interventions into routine care. Clinicians should consider recommending or prescribing digital tools that are compatible with wearable technologies, especially for individuals with greater illness burden. Additionally, the finding that lower health‐related quality of life (EQ‐5D‐5L index) was associated with higher utilization suggests that using objective measures of clinical need to assess health‐related quality of life as opposed to relying on subjective perceptions of well‐being could provide more insight into clinically relevant health burden. Given that the codes for this measurement are region specific, using the EQ‐5D‐5L index in clinical practice could provide context‐specific and objective measurement of health‐related quality of life among individuals with BD.

At the same time, the study identified notable disparities in the use of digital health resources across different demographic groups, with lower engagement observed among those identifying as male and individuals diagnosed later in life. Policy makers should invest in implementing gender‐sensitive design approaches, and healthcare providers should prioritize early diagnosis so as to target individuals with delayed or late diagnosis when recommending or prescribing digital health resources as a treatment option. Furthermore, healthcare providers require enhanced training and awareness of available digital health resources to effectively recommend appropriate tools. In Germany, the acceptance of digital health resources among healthcare providers has been linked to the availability of reliable information on their effectiveness, safety, and impact on care [65]. Also, studies showed clinicians to be rather skeptical in engaging with digital health resources [66], which can be in contrast to their patients' preferences and therefore hinder insights and opportunities for illness management. Interestingly, over 40% of participants reported a positive influence of the use of digital health resources on their decision making. Due to the chronic nature with sometimes complex decisions regarding pharmacological management like handling long‐term Lithium therapy, digital health resources could empower patients in their decision making.

With nearly half of all approved digital health applications in Germany (DiGAs) (27 out of 55) designed for the treatment of mental illnesses [46], there remains a lack of interventions specifically tailored to BD. Developing evidence‐based, BD‐specific digital interventions, particularly those integrating wearable technologies and addressing clinically relevant needs, may therefore represent an important step towards improving care for this population.

### 4.8 Strengths and Limitations

This study possesses several strengths, including a large sample size (*N* = 213) and the application of a well‐established theoretical framework, the AHUA, to guide the analysis. To our knowledge, this is the first time applying the Andersen model in the context of digital health care use among individuals with BD. The inclusion of a diverse sample recruited online further captures a broad demographic range.

However, certain limitations must be acknowledged. A large proportion of the survey respondents were highly educated, which introduces selection bias, biasing the generalizability of the findings. Additionally, diagnoses in the study were self‐reported rather than confirmed through standardized clinical interviews, which may introduce variability and potential misclassification within the sample. However, prior research suggests that self‐reported BD diagnoses can exhibit acceptable validity; for example, one study found that 93% of a randomly selected sample (*n* = 100) met the criteria for a lifetime BD spectrum diagnosis when assessed through structured clinical interviews [67]. Nonetheless, the possibility of inaccurate reporting, including misclassification or response bias in online samples, cannot be fully excluded. Furthermore, reliance on self‐reported data for the use of digital health care resources and the study's cross‐sectional design constrain the ability to establish causal relationships. Future research should therefore aim to replicate these findings by using longitudinal designs and clinically verified diagnoses.

Regarding the HLS‐DIGI‐DD instrument, moderate reliability suggests that the six‐item scale may not fully capture digital healthcare utilization. While this level of reliability was sufficient for an exploratory study, further refinement of the instrument is warranted in future research. Potential improvements include revising or expanding items related to digital health‐seeking behaviors, incorporating measures of artificial intelligence‐based resources, expanding response scales, and enhancing item clarity. These refinements could improve the instrument's reliability and provide more precise assessments of digital health utilization.

Finally, in the hierarchical multiple linear regression analysis, the role of cognitive functioning as operationalized by concentration difficulties was examined as a factor influencing digital health utilization, but did not result in a significant effect. Additionally, the interaction between concentration difficulties and wearables was examined but did not yield a significant combined effect in influencing digital health utilization. Instead, only wearables emerged to be independently associated with digital health utilization in the model. Future research could explore whether a more nuanced interaction model, potentially incorporating instruments specific for assessing cognition such as the Montreal Cognitive Assessment or the Mini‐Mental State Examination, or a larger sample size, might reveal alternative patterns of association.

### 4.9 Future Research Directions

Future research should explore interventions aimed at increasing digital health utilization among underrepresented groups, such as older and male individuals and those diagnosed later in life. Longitudinal studies are needed to assess how digital health utilization evolves over time and to identify factors that sustain engagement. While digital health experience does facilitate decision‐making, its role is not absolute. Users still encounter barriers such as information overload, trust issues, and health literacy challenges. Future research should explore how digital health literacy training, AI‐driven personalization, and stronger provider‐patient integration can enhance decision‐making confidence and inform the design of more accessible and effective resources.

## 5 Conclusion

This study provides valuable insights into the high utilization of digital health resources among individuals with BD in Germany. The findings demonstrate that utilization is primarily driven by enabling factors, particularly wearable ownership, and objective indicators of clinical need, rather than by general sociodemographic characteristics or subjective health perceptions. In particular, individuals with lower health‐related quality of life and earlier age of diagnosis were more likely to engage with digital health tools, suggesting that utilization is closely linked to measurable health burden and longer‐term illness management. While digital tools such as wearable devices appear to support self‐management, digital interactions with healthcare providers remain underutilized, indicating an important gap in current service delivery. These findings highlight the need to move beyond general access and focus on integrating digital tools into care pathways, particularly for individuals with higher clinical need. Promoting earlier diagnosis and facilitating access to clinically relevant, evidence‐based digital interventions, especially those incorporating wearable technologies, may enhance engagement and support long‐term management of bipolar disorder. Overall, this study underscores the importance of adopting a theory‐driven approach to understanding digital health utilization and provides a foundation for the development of more targeted and equitable digital health solutions.

## Author Contributions

**Linda Kokwaro:** methodology, investigation, software, validation, data curation, visualization, writing – original draft, writing – reviewing and editing. **Helena Krüger:** methodology, investigation, writing – reviewing and editing. **Dennis Stratmann:** methodology, investigation, writing – reviewing and editing. **Daniel Schulze:** methodology, investigation, writing – reviewing and editing. **Daniel Fürstenau:** supervision, writing – reviewing and editing. **Eva Friedel:** writing – reviewing and editing. **Esther Quinlivan:** writing – reviewing and editing. **Surjo R. Soekadar:** supervision, writing – reviewing and editing. **Sonia Lech:** methodology, investigation, software, validation, writing – original draft preparation, supervision, writing – reviewing and editing. **Stefanie Schreiter:** conceptualization, writing – original draft preparation, supervision, writing – reviewing and editing.

## Funding

This work was supported by Einstein Center for Neurosciences Berlin.

## Conflicts of Interest

The authors declare no conflicts of interest.

## Supporting information


**Table S1:** Overview of variables and measures used in the survey.
**Table S2:** Summary of the CFA results of the HL‐DIGI‐DD instrument comparing the ML and WLSMV estimators, including fit indices.
**Table S3:** Summary of the CFA results of the HL‐DIGI‐DD instrument showing reliability test statistics, fit indices and factor loadings.
**Table S4:** Hierarchical multiple linear regression model examining pre‐disposing, enabling and need factors associated with digital health resource utilization (step 5 only).
**Figure S1:** Plot showing the whole sample (*N* = 213) categorized into users (orange) and non‐users (blue) accessing different BD‐specific digital health resources including websites (Users: 92% (*N* = 192), Non‐users: 7.2% (*N* = 15)), social media and online forums (Users: 82.7% (*N* = 163), Non‐users: 17.3% (*N* = 34)), smartphone health apps (Users: 53.8% (*N* = 92), Non‐users: 46.2% (*N* = 79)), other information sources (Users: 76.9% (*N* = 140), Non‐users: 23.1% (*N* = 42)), digital interaction (Users: 61.2% (*N* = 101), Non‐users: 38.8% (*N* = 64)), digital health devices (Users: 53.1% (*N* = 93), Non‐users: 46.9% (*N* = 82)). *X* axis shows the percentage (%) of respondents and the *y* axis shows the categories of digital health resources.
**Figure S2:** Plot showing users only and their frequency of accessing BD‐specific digital health resources including websites (Daily: 12% (*N* = 23), Weekly: 22.4% (*N* = 43), Rarely: 65.6% (*N* = 126)), social media and online forums (Daily: 20.2% (*N* = 33), Weekly: 22.1% (*N* = 36), Rarely: 57.7% (*N* = 94)), smartphone health apps (Daily: 41.3% (*N* = 38), Weekly: 13% (*N* = 12), Rarely: 45.7% (*N* = 42)), other information sources (Daily: 5.7% (*N* = 8), Weekly: 15.7% (*N* = 22), Rarely: 78.6% (*N* = 110)), digital interaction (Daily: 1% (*N* = 1), Weekly: 10.9% (*N* = 11), Rarely: 88.1% (*N* = 89)), digital health devices (Daily: 38.7% (*N* = 36), Weekly: 20.4% (*N* = 19), Rarely: 40.9% (*N* = 38)). Frequency of use was classified in the following categories: rarely (green), weekly (orange) and daily (purple). *X* axis shows the count and *y* axis shows the categories of digital health resources.

## Data Availability

All data generated or analyzed for this study are available within the paper and its associated Supporting Information files. All other data and the codes used for this study are available from the corresponding author upon reasonable request. The data are not publicly available due to privacy or ethical restrictions.

## Endnotes

^1^
Throughout this paper, digital health resources refer to health‐related websites, social media and online forums, smartphone apps, wearable devices, and remote communication with healthcare providers.
