# Supplementary material for: Determinants of Digital Health Resource Utilization Among Individuals With Self‐Reported Bipolar Disorder in Germany—A Cross‐Sectional Study Based on the Andersen Model
Source: Bipolar Disord. 2026 Jun 9;28(4):e70128. doi: 10.1111/bdi.70128 (PMC13248575; doi:10.1111/bdi.70128)
Supplement: Supplementary file 1 — Table S1: Overview of variables and measures used in the survey. Table S2: Summary of the CFA results of the HL‐DIGI‐DD instrument comparing the ML and WLSMV estimators, including fit indices. Table S3: Summary of the CFA results of the HL‐DIGI‐DD instrument showing reliability test statistics, fit indices and factor loadings. Table S4: Hierarchical multiple linear regression model examining pre‐disposing, enabling and need factors associated with digital health resource utilization (step 5 only). Figure S1: Plot showing the whole sample (N = 213) categorized into users (orange) and non‐users (blue) accessing different BD‐specific digital health resources including websites (Users: 92% (N = 192), Non‐users: 7.2% (N = 15)), social media and online forums (Users: 82.7% (N = 163), Non‐users: 17.3% (N = 34)), smartphone health apps (Users: 53.8% (N = 92), Non‐users: 46.2% (N = 79)), other information sources (Users: 76.9% (N = 140), Non‐users: 23.1% (N = 42)), digital interaction (Users: 61.2% (N = 101), Non‐users: 38.8% (N = 64)), digital health devices (Users: 53.1% (N = 93), Non‐users: 46.9% (N = 82)). X axis shows the percentage (%) of respondents and the y axis shows the categories of digital health resources. Figure S2: Plot showing users only and their frequency of accessing BD‐specific digital health resources including websites (Daily: 12% (N = 23), Weekly: 22.4% (N = 43), Rarely: 65.6% (N = 126)), social media and online forums (Daily: 20.2% (N = 33), Weekly: 22.1% (N = 36), Rarely: 57.7% (N = 94)), smartphone health apps (Daily: 41.3% (N = 38), Weekly: 13% (N = 12), Rarely: 45.7% (N = 42)), other information sources (Daily: 5.7% (N = 8), Weekly: 15.7% (N = 22), Rarely: 78.6% (N = 110)), digital interaction (Daily: 1% (N = 1), Weekly: 10.9% (N = 11), Rarely: 88.1% (N = 89)), digital health devices (Daily: 38.7% (N = 36), Weekly: 20.4% (N = 19), Rarely: 40.9% (N = 38)). Frequency of use was classified in the following categories: rarely (green), weekly (orange) [file BDI-28-0-s001.docx]

Supplementary Materials

**Supplementary Table 1**

*Overview of variables and measures used in the survey*

| Section of the Andersen Model | Variable | Measurement | ANSWER  OPTIONS |
| --- | --- | --- | --- |
| Predisposing Factors | age | Self-developed questionnaire | Years |
|  | gender | Self-developed questionnaire | Male  Female  Diverse |
|  | education level | Self-developed questionnaire | Years of education |
|  | employment status | Self-developed questionnaire | Employed, working full-time  Employed, part-time  Self-employed  Student  Training/retraining  Job seeker, receiving unemployment benefit I or II  Family support (e.g. from spouse)  Pension  Regular old-age pension  Reduced earning capacity pension |
| NEED Factors | age of diagnosis | Self-developed questionnaire | Years |
|  | age of symptom onset | Self-developed questionnaire | Years |
|  | Occurrence of the last affective episode  EQ-5D-5L | Self-developed questionnaire  EQ-5D-5L | Depressive,  Hypomanic,  Manic  Likert Scale |
| Enabling Factors | Digital Health Literacy  (type of skill,  competence level)  Having a smartphone  Having a wearable | HL-DIGI, HL-DIGI-INT | Yes/No  Yes/No |
| Other Variables |  |  |  |
|  | Digital Health Utilization  (resource type,  frequency of use)  Concentration  Difficulties | HL-DIGI-DD  QIDS-SR |  |
|  | Symptomatology | QIDS-SR, ASRM |  |

**Supplementary Table 2**

*Summary of the CFA results of the HL-DIGI-DD instrument comparing the ML and WLSMV estimators, including fit indices*

| **Fit Index** | **ML Model** | **WLSMV Model** |
| --- | --- | --- |
| CFI | \| 0.82 \| \| --- \| | \| 0.97 \| \| --- \| |
| TLI | 0.70 | 0.96 |
| RMSEA | 0.12 | 0.07 |
| SRMR | 0.07 | 0.07 |

*Note.*Confirmatory Factor Analysis conducted to examine the underlying structure of the six items measuring Digital Health Resource Utilization. The analysis was first performed using the 2 different estimators : ML (Maximum Likelihood) for robustness and WLSMV (Weighted Least Squares Mean and Variance Adjusted) to account for ordinal responses. The CFA model demonstrated excellent fit indices, indicating that the six items collectively measure a single latent construct, Digital Health Resource Utilization. WLSMV fits much better i.e CFI (Comparative Fit Index) =0.97 ; excellent fit (CFI > 0.95), TLI (Tucker-Lewis Index) =0.96 ; excellent fit (TLI > 0.95), RMSEA (Root Mean Square Error of Approximation) =0.07; acceptable fit (RMSEA < 0.08) and SRMR (Standardized Root Mean Square Residual) =0.07 ; Good fit (SRMR < 0.08).

**Supplementary Table 3**

*Summary of the CFA results of the HL-DIGI-DD instrument showing reliability test statistics, fit indices and factor loadings*

| **Latent Variable** | **Item** | **Estimate** | **Std.Estimate** | **z-value** | **p-value** | **significance** |
| --- | --- | --- | --- | --- | --- | --- |
| Digital Health  ResourceUse | Health-related websites | 1.00 | 0.73 | - | - |  |
|  | Social media & online forums | 1.19 | 0.85 | 4.60 | 0.00 | *** |
|  | Digital health devices | 1.79 | 1.30 | 2.37 | 0.02 | * |
|  | Smartphone health apps | 1.92 | 1.40 | 2.21 | 0.03 | * |
|  | Digital interaction | 1.02 | 0.74 | 2.73 | 0.01 | ** |
|  | Other information sources | 1.74 | 1.27 | 3.35 | 0.00 | ** |

*Note** p < .05. ** p < .01. *** p < .001. The significance levels used take the adjustment of the p-values into account, which were divided by the respective number of independent variables contained in the model using Bonferroni correction

|  |
| --- |
|  |
|  |
|  |
|  |

**Supplementary Table 4**

*Hierarchical multiple linear regression model examining pre-disposing, enabling and need factors associated with digital health resource utilization (step 5 only)*

| *Variable* | **B [95% CI]** | **β** |
| --- | --- | --- |
| **Predisposing factors** |  |  |
| Age | 0.01 [-0.00, 0.02] | .13 |
| Gender | 0.39^*^ [0.08, 0.69] | .18^*^ |
| Employment | 0.11 [-0.43, 0.66] | .03 |
| Education | -0.11 [-0.25, 0.04] | -.10 |
| **Enabling factors** |  |  |
| Smartphone ownership | 0.47 [-0.55, 1.49] | .06 |
| Wearable ownership | 0.89^***^ [0.59, 1.20] | .41^***^ |
| DHL score | 0.00 [-0.00, 0.01] | .05 |
| **Cognition / interaction** |  |  |
| Concentration | 0.03 [-0.19, 0.25] | .03 |
| Wearable × concentration | 0.01 [-0.35, 0.38] | .01 |
| **Need factors** |  |  |
| Last affective episode | -0.10 [-0.22, 0.03] | -.12 |
| Age of diagnosis | -0.02^*^ [-0.04, -0.00] | -.21^*^ |
| Age of symptom onset | 0.00 [-0.01, 0.01] | .00 |
| EQ-5D-5L VAS (10-point) | 0.07 [-0.02, 0.15] | .15 |
| EQ-5D-5L index | -1.26^**^ [-2.13, -0.38] | -.28^**^ |
| **Model summary** |  |  |
| R² | .31 |  |
| ΔR² (final step) | .08^**^ |  |

*Note*. N = 168 . Results of the final step (step 5) of a hierarchical multiple linear regression model. Cells report unstandardized coefficients (B) with 95% confidence intervals in brackets and standardized coefficients (β). R^2^ = explained variance; ΔR^2^ = change in explained variance. Values are rounded to two decimal places. *p* < .05*, *p* < .01**, *p* < .001***.

**Supplementary Figure 1**


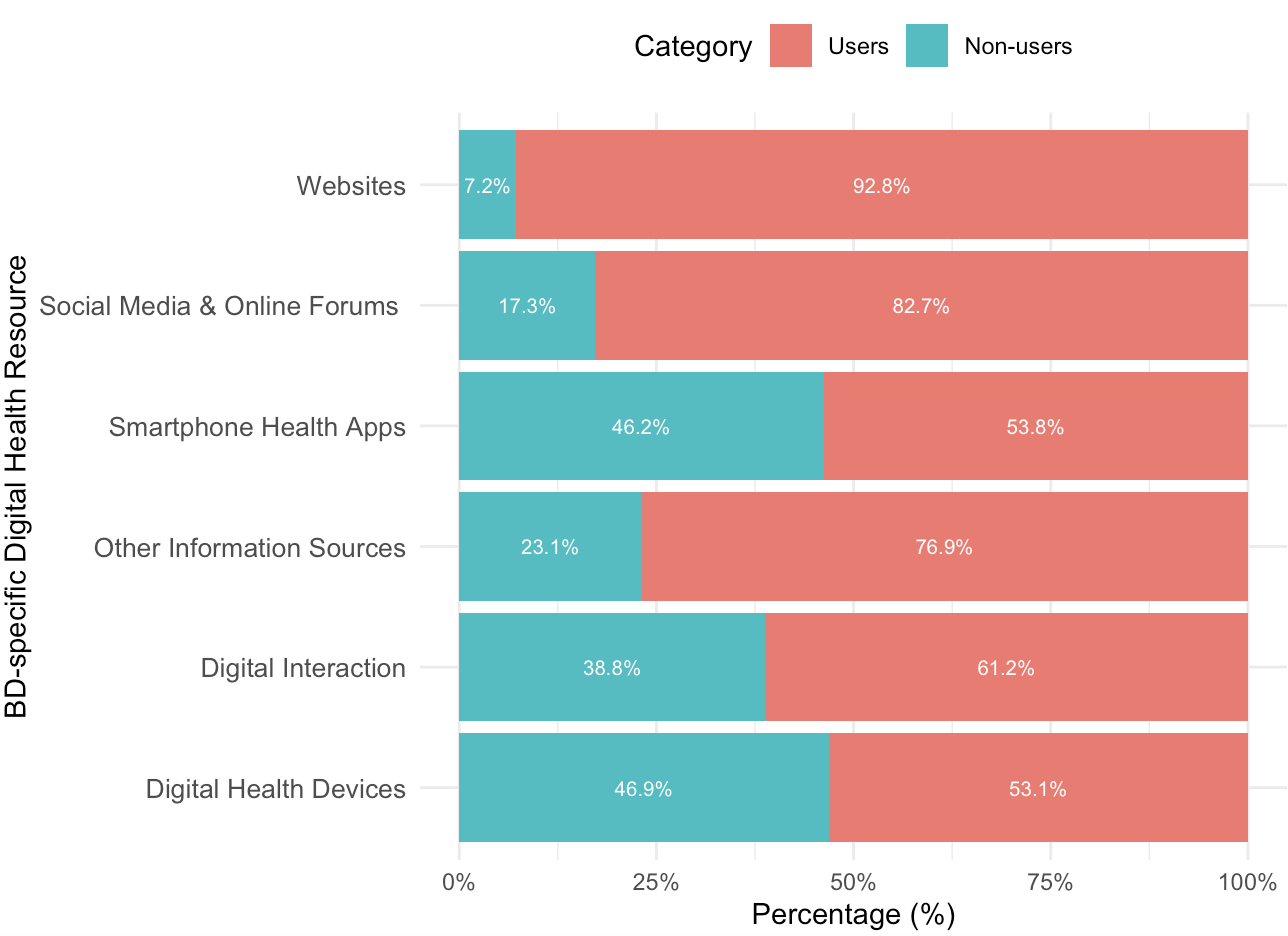


**Supplementary Figure 1.**Plot showing the whole sample (N=213) categorized into users (orange) and non-users (blue) accessing different BD-specific digital health resources including websites (Users: 92 % (N = 192), Non-users: 7.2 % (N = 15)), social media and online forums (Users: 82.7 % (N = 163), Non-users: 17.3 % (N = 34)), smartphone health apps (Users: 53.8 % (N = 92), Non-users: 46.2% (N = 79)), other information sources Users: 76.9 % (N = 140), Non-users: 23.1 % (N = 42)), digital interaction Users: 61.2 % (N = 101), Non-users: 38.8 % (N = 64)), digital health devices (Users: 53.1 % (N = 93), Non-users: 46.9 % (N = 82)). X axis shows the percentage (%) of respondents and the y axis shows the categories of digital health resources.

**Supplementary Figure 2**


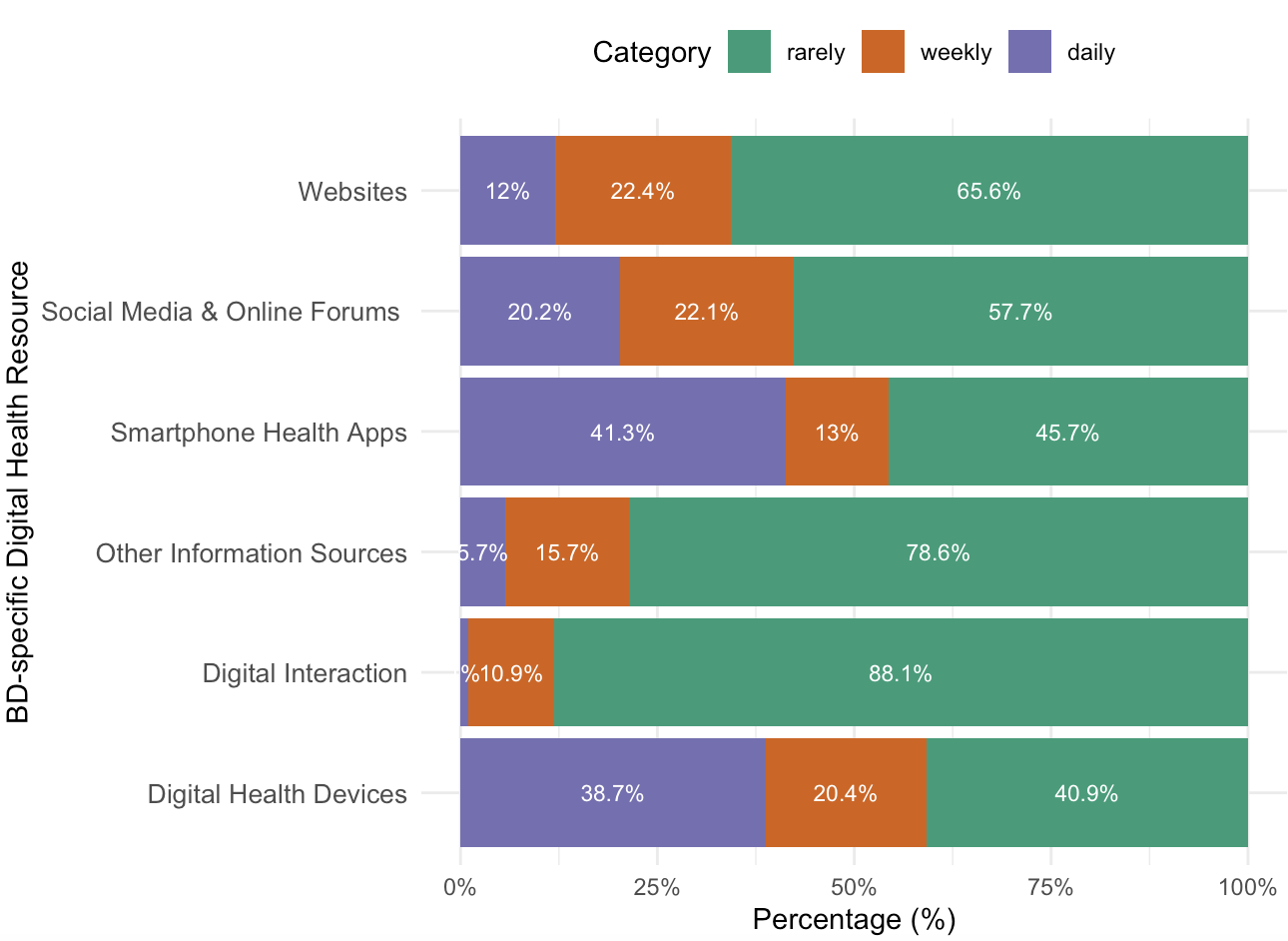


**Supplementary Figure 2***.* Plot showing users only and their frequency of accessing BD-specific digital health resources including websites (Daily : 12 % (N = 23), Weekly: 22.4 % (N = 43), Rarely: 65.6 % (N = 126)), social media and online forums (Daily : 20.2 % (N = 33), Weekly: 22.1 % (N = 36 ), Rarely: 57.7 % (N = 94 ), smartphone health apps (Daily : 41.3 % (N = 38), Weekly: 13 % (N = 12), Rarely: 45.7 % (N = 42)), other information sources (Daily : 5.7 % (N = 8 ), Weekly: 15.7 % (N = 22 ), Rarely: 78.6 % (N = 110 )), digital interaction (Daily : 1 % (N = 1 ), Weekly: 10.9 % (N = 11), Rarely: 88.1 % (N = 89)), digital health devices (Daily : 38.7 % (N = 36 ), Weekly: 20.4 % (N = 19 ), Rarely: 40.9 % (N = 38)). Frequency of use was classified in the following categories: rarely (green), weekly (orange) and daily (purple). X axis shows the count and y axis shows the categories of digital health resources.
